# Retraction: Silencing of LncRNA PVT1 inhibits the proliferation, migration and fibrosis of high glucose-induced mouse mesangial cells via targeting microRNA-93-5p

**DOI:** 10.1042/BSR-20194427_RET

**Published:** 2020-06-25

**Keywords:** diabetic nephropathy, LncRNA- PVT1, miR-93-5p, PTEN, PI3K/Akt/mTOR pathway, mouse mesangial cells

The article “Silencing of LncRNA PVT1 inhibits the proliferation, migration and fibrosis of high glucose-induced mouse mesangial cells via targeting microRNA-93-5p” (*Bioscience Reports* (2020) 40(5), DOI: 10.1042/BSR20194427) is being retracted at the request of the Editor-in-Chief and the Editorial Board following receipt of a notification from a reader, alerting the Editorial Board to manipulation in Figure 2C, as well as duplicated images in Figures 2B, 4B, 7B and 7D.

The authors have been contacted in regard to the retraction, and disagree with the Journal's decision to retract the article; however, the Editorial Board stand by the decision to retract given the concerns with the figures.

